# The shifting burden of gastrointestinal and liver diseases in Southeast Asia and effective control strategies

**DOI:** 10.2478/abm-2025-0027

**Published:** 2025-10-31

The burden of gastrointestinal (GI), liver, and pancreatic diseases has undergone a significant shift worldwide [1, 2]. Some problems, such as infectious diarrhea and parasitic diseases, have declined particularly in high-income countries; they pose a significant problem in many low-income countries [3].

The changes in aging profiles and fertility have given rise to an ever-changing demographic profiles [4, 5]. Improvements in sanitation, living conditions, and infectious disease control have led to increased life expectancy. However, new challenges have emerged, including lifestyle and metabolic risk factors such as obesity and sedentary behavior, which are contributing to the growing prevalence of GI and liver diseases—independent of cardio-metabolic conditions [6]. Metabolic dysfunction-associated steatotic liver disease (MASLD)—formerly known as non-alcoholic fatty liver disease (NAFLD)—is rising in prevalence across many areas. MASLD is associated with cirrhosis and hepatocellular carcinoma [7]. This has manifested in the disparities of these conditions between high- and low-income countries [8].

Although infectious GI and parasitic diseases have declined, hepatitis B (HBV) and hepatitis C (HCV) infections have increased the prevalence of hepatocellular carcinoma [9]. Behavioral risks for HBV and HCV primarily revolve around behaviors that can transmit the virus through bodily fluids, commonly seen in risk behaviors such as injecting drug users, unprotected sex due to the lack of knowledge about transmission, and risky sexual practices. These have implications for the changing profiles of GI and liver diseases in the population, including the elderly [10]. These findings call for a need for modification of high-risk lifestyle behaviors in the primary prevention of chronic liver disease in the general population.

Some drivers of change are urbanization and health care access in regions of the world. Processed diets, alcohol, and tobacco use in urban areas can be detrimental to liver and metabolic diseases. Access to proper diagnostics and the possibilities of early treatments are available for high-income communities. The mortality rate for most GI diseases was sensitive to urbanization, and control of external risk factors could lead to the conversion of most GI diseases [11].

In this issue, Suenghataiphorn et al. [12] have summarized the shifting burden of GI and liver diseases across 11 Southeast Asian nations. They concluded that Southeast Asia confronts a complex and changing burden of GI and liver diseases. Although successes against most infectious diseases are commendable, there has been a growing and challenging threat from noncommunicable and lifestyle diseases. These include GI cancers and metabolic liver diseases linked to hepatitis and MASLD. They argued that since countries have different rates of urbanization and health care access, there is an urgent need for tailored national strategies focused on prevention (e.g., vaccination, nutrition policies, and alcohol moderation), early diagnosis, and improved equitable access to effective treatments, which cannot be overemphasized [13–15].
